# Venous thromboembolism events among RA patients

**DOI:** 10.31138/mjr.30.1.38

**Published:** 2019-03-28

**Authors:** Ribhi Mansour, Shir Azrielant, Abdulla Watad, Shmuel Tiosano, Yarden Yavne, Doron Comaneshter, Arnon D. Cohen, Howard Amital

**Affiliations:** 1 Department of Medicine ‘B’, Sheba Medical Center, Israel; 2 Zabludowicz Center for Autoimmune Diseases, Sheba Medical Center, Israel;; 3 Sackler Faculty of Medicine, Tel-Aviv University, Tel Aviv, Israel,; 4 Department of Quality Measurements and Research, Chief Physician’s Office, Clalit Health Services, Tel Aviv, Israel,; 5 Siaal Research Center for Family Medicine and Primary Care, Faculty of Health Sciences, Ben-Gurion University of the Negev, Beer-Sheva, Israel

**Keywords:** Rheumatoid arthritis, deep vein thrombosis, venous thromboembolism, C-Reactive Protein, thrombosis, autoimmune diseases

## Abstract

**Background:**

Rheumatoid arthritis (RA) is associated with an increased risk for venous thromboembolism. However, so far, relatively few and small size-based studies have been conducted. We aimed to investigate the link between RA and venous thromboembolism utilizing a large sample of subjects originating from a large data base.

**Materials and methods:**

The study was performed utilizing the medical database of Clalit Health Services, the largest healthcare provider in Israel. We enrolled all patients with RA and age- and gender-matched controls. Chi-square and t-tests were used for univariate analysis and a logistic regression model was used for a multivariate analysis. RA patients were compared to controls regarding the proportion of venous thromboembolic events (defined as deep vein thrombosis, pulmonary embolism or both). Multivariate logistic regression was employed to assess factors associated with thromboembolic events.

**Results:**

The study included 11,782 patients with RA and 57,973 age- and gender-matched controls. RA patients had a higher rate of venous thromboembolism events compared with controls (6.92% vs. 3.18%, respectively, p<0.001). RA and mean C-reactive protein levels were found to be independently associated with the proportion of thromboembolic events (OR 2.27 for RA and 1.07 for each 1 mg/dL increment of mean C-reactive protein, respectively).

**Conclusion:**

RA and C-reactive protein levels are independently associated with venous thromboembolic events. Physicians should be aware of such findings and have a lower threshold for suspecting detecting such events in patients with RA, mainly those with mean high levels of C-reactive protein.

## INTRODUCTION

Rheumatoid arthritis (RA) is a chronic and systemic autoimmune disease, characterized by a symmetrical inflammation of the joints; RA is considered common in comparison to other autoimmune diseases, and its prevalence among Northern Europeans and North Americans is estimated to be 0.5–1%.^1,2^

RA has many extra articular manifestations; cutaneous, salivary glands, ophthalmic, pulmonary and neurological manifestations are often reported.^2^ Life-threatening complications of pulmonary embolism, excessive morbidity and mortality related to thromboembolism and cardiovascular disease are the main factors that contribute to the detrimental burden carried by patients with RA which significantly affects their longevity.^3^ Interestingly, many RA patients also have higher rates of traditional risk factors for atherosclerosis, of which smoking takes a lead and many of these patients are also less mobile due to the musculoskeletal outcomes of the disease. All these factors converge to accelerated inflammation, in which the coagulation system also plays a role.^2–4^

Several studies have shown that the RA inflammatory state increases the concentration of different pro-coagulant and thrombolytic factors.^5,6^ Mounting evidence suggests that the innate immune system and coagulation system share a common evolutionary origin, and extensive crosstalk exists between inflammatory cytokines and coagulation factors. Hence, the activation of the inflammatory cytokine networks may also induce prothrombotic conditions such as endothelial dysfunction, tissue factor (TF) overexpression, and inhibition of fibrinolysis and protein C.^7^

Our goal in this study was to assess the association between RA and venous thromboembolic events (VTE), using the database of the largest healthcare provider in Israel.

## METHODS

### Ethical approval

This study was approved by an institutional ethics committee in Soroka Hospital, Beer-Sheba, Israel. It was exempted by the ethics committee from signing informed consent forms.

### Patients selection

This study is one of a series of explorative and analytic studies based on the chronic disease registry of CHS (Clalit Health Services), the largest healthcare provider in Israel that covers over 4.4 million enrollees, grossly half of the Israeli population.

CHS has a comprehensive computerized database with continuous real-time input from pharmaceutical, medical and administrative computerized operating systems. The database contains a chronic diseases registry, based on data from hospital discharge notes for inpatients and primary care and expert physicians’ reports for outpatients. The study was designed as a cross-sectional analysis. All RA patients in CHS’s database were included in the study, as well as five age- and gender-matched controls for each RA patient. Data for each patient included age, gender, socioeconomic status (SES), smoking status, body mass index (BMI), previous diagnosis of VTE (defined as previous diagnosis of deep vein thrombosis “DVT”, pulmonary embolism “PE” or both), and previously documented levels of CRP. RA patients were defined as such when they had at least one documented diagnosis of RA, either by community physician or hospital discharge note. DVT/PE diagnosis was defined in a similar fashion. Due to the structure of the database, it was not possible to determine the etiology of each disorder. However, the validity of the diagnoses in the registry was found to be high in previous studies.^1,8–11^

CRP levels were represented by a mean value of all measured laboratory CRP levels for each participant in blood exams from 2002–2013 (regardless of time and clinical setting within which the test was taken).

### Statistical analysis

We used student’s t-test to test difference between continuous variables and Chi-Square for categorical variables. Univariate analysis was conducted to assess the distribution of different study covariates between patients with and without VTE.

A logistic regression model was used to test the association between RA and VTE while adjusting for age, gender, SES, smoking status and BMI. An additional model included all of the aforementioned variables, as well as CRP levels. Point estimates are presented with 95% confidence interval.

Statistical analysis was preformed using R Statistical Software (version 3.2.2; R Foundation for Statistical Computing, Vienna, Austria).

## RESULTS

The study included 11,782 patients with RA, and 57,973 age- and gender-matched controls (*Table 1*). As expected by the epidemiology of RA disease, most of the patients in our cohort were females (about 77% in both groups). Of note, smoking was more frequent among RA patients in comparison with controls, as was high BMI.

**Table 1.** Basic characteristics of study population.

**Characteristic**	**Controls**	**RA patients**	**p value**
	N=57,973	N=11,782	
**Age, Mean±SD**	60.8±17.0	61.1±17.0	0.174
**Gender: Male**	13384 (23.1%)	2679 (22.7%)	0.413
**BMI, Mean±SD**	28.0±6.58	28.2±6.21	0.003
**SES:**			
**Low**	22657 (39.2%)	4505 (38.3%)	Ref.
**Medium**	22831 (39.5%)	4816 (41.0%)	0.009
**High**	12334 (21.3%)	2438 (20.7%)	0.831
**Smoking**	16671 (28.8%)	3865 (32.8%)	<0.001
**CRP, Mean±SD**	0.93±1.94	1.25±1.43	<0.001
**VTE**	1841 (3.18%)	815 (6.92%)	<0.001

SD: Standard deviation; BMI: Body mass index, kg/m^2^; SES: Socioeconomic status; CRP (years 2002–2013), reference range: 0–0.5 mg/dL; VTE: Venous thromboembolism, defined as DVT, PE or both.

Our study revealed that RA patients had a higher rate of VTE diagnosis compared with controls (6.92% vs. 3.18%, respectively, p<0.001). of these events in the RA group, 704 were diagnosed with DVT (6%) and 160 with PE (1.4%).

Univariate analysis (*Table 2*) has shown that RA patients who experienced VTE were older, more likely to be female and had a higher BMI and a higher mean CRP value. RA patients had a high risk of VTE (OR 2.27, CI 2.80–2.47). Surprisingly, smoking was not found to be associated with a history of VTE.

**Table 2.** Comparison between patients with and without VTE.

**Characteristic**	**Subjects without VTE**	**Subjects with VTE**	**OR**	**p value**
	N=67099	N=2656		
**Age, Mean±SD**	60.5±17.0	69.5±13.4	1.04 [1.04;1.04]	<0.001
**Gender:**				
**Female**	51507 (76.8%)	2185 (82.3%)	Ref.	Ref.
**Male**	15592 (23.2%)	471 (17.7%)	0.71 [0.64;0.79]	<0.001
**BMI, Mean±SD**	28.0±6.51	30.3±6.27	1.05 [1.04;1.06]	<0.001
**SES:**				
**Low**	26141 (39.1%)	1021 (38.6%)	Ref.	Ref.
**Medium**	26565 (39.7%)	1082 (40.9%)	1.04 [0.96;1.14]	0.346
**High**	14228 (21.3%)	544 (20.6%)	0.98 [0.88;1.09]	0.695
**RA**	10967 (16.3%)	815 (30.7%)	2.27 [2.08;2.47]	<0.001
**Smoking**	19788 (29.5%)	748 (28.2%)	0.94 [0.86;1.02]	0.140
**CRP, Mean±SD**	1.03±1.77	1.32±2.16	1.07 [1.05;1.09]	<0.001

SD: Standard deviation; BMI: Body mass index, kg/m^2^; SES: Socioeconomic status; CRP (years 2002–2013), reference range: 0–0.5 mg/dL; VTE: Venous thromboembolism, defined as DVT, PE or both.

*Table 3* (model 1 and 2) demonstrates an independent association of VTE with increasing age and BMI, as well as female gender and RA. In model 2, it is shown that CRP levels were found to be linearly associated with the odds having VTE, regardless of RA.

**Table 3.** Multivariate logistic regression models (with and without CRP) assessing covariates independently associates with VTE.

	**Model 1 (without CRP) OR**	**Model 2 (with CRP) OR**
**Age, per year**	1.04 (1.04–1.04)^*^	1.03 (1.03–1.04)^*^
**Gender: Male vs. Female**	0.77 (0.69–0.85)^*^	0.81 (0.7–0.93)^*^
**BMI, per 1 kg/m^2^**	1.05 (1.04–1.05)^*^	1.05 (1.04–1.06)^*^
**SES: Medium vs. Low**	0.94 (0.86–1.03)	0.92 (0.81–1.04)
**SES: High vs. Low**	0.91 (0.82–1.02)	0.93 (0.81–1.07)
**Smoking**	1.05 (0.95–1.15)	1.08 (0.96–1.21)
**RA**	2.23 (2.05–2.43)^*^	1.60 (1.44–1.78)^*^
**CRP, per 1 mg/dl**	-	1.06 (1.03–1.08)^*^

* p<0.05

## DISCUSSION

Cardiovascular disorders are well documented to coexist in RA patients; this association is believed to be highly related with the degree of the inflammatory process. Studies to date have focused mainly on arterial atherosclerotic manifestations such as myocardial infarction and increased carotid artery stenosis, as targets for diagnosis and therapeutic interventions.^1,12–15^ Studies on the role of RA and its systemic inflammation in VTE are not as common and are now emerging.

Our data exhibited a significant association between RA and VTE, showing a twofold increase in the frequency of VTE among RA patients. Our results match those of other reports. Chung et al.^16^ have published a population-based ten-year-cohort study from Taiwan. Out of the 23.74 million people in their cohort, there were 29,238 RA patients (77% women, mean age of 52.4 years), matched with 116,952 controls. The risk for developing DVT and PE in RA patients was increased by 3.36-fold and 2.07-fold respectively, compared to controls, after adjusting for age, gender and comorbidities. Other studies, based on hospitalized patients, also support the concept that RA patients have an increased risk for VTE.^17,18^ In the Copenhagen General Population Study, conducted between the years 2004 and 2012, researchers measured the concentration of immunoglobulin M (IgM) type RF (Rheumatoid factor) in patients without autoimmune rheumatic disease or VTE. The main outcome was the incidence of DVT. In a total of 368,381 person-years, 670 individuals developed DVT. An RF concentration higher than 110 IU/mL showed the strongest association with DVT with multivariable adjusted hazard ratios of 9.0 (95% CI 3.1–26) for 1-year follow-up, 4.3 (2.2–8.5) for 5-year follow-up, and 3.1 (1.7–5.6) for up to 32 years of follow-up.^19^

Our data, similarly to previous studies, may suggest that there should be a role for thromboprophylaxis in immune mediated diseases such as RA. From an economical aspect, VTE bears a great burden on the health system. Predicted costs of medical care were found to be 2.5-fold higher for patients with VTE related to current or recent hospitalizations for acute illness (62,838 US$) when compared to hospitalized patients without VTE (24,464 US$; P <0.001).^20^ These finding highlight even further the need for appropriate prophylaxis, as well as the need to add rheumatic conditions and RA in particular to different risk models assessing the chances of developing VTE during hospitalization.

The Padua Prediction Score was created to assess the risk for VTE in hospitalized patients, and determine their need for prophylaxis. Although the Padua Prediction Score identifies acute rheumatologic disorders as increased risk states for VTE, their importance in the scale is relatively low.^21^ The presented results may imply that the relative impact of a current inflammatory rheumatic condition should be assessed differently suggesting a more substantial contribution to the risk of VTE.

To date, the underlying pathophysiologic pathway for the association between RA and the hypercoagulable state is not fully understood, however, several steps in the inflammatory process have been linked to hypercoagulability.^22–24^ Hypercoagulability could be induced by inflammation, for example via cytokine-induction of tissue factor (TF) expression, endothelial dysfunction, inhibition of the protein C system and inhibition of fibrinolysis.^22–25^ Inflammatory mediators, like CRP, tumour necrosis factor-alpha (TNFα), interleukin-6 (IL-6) as well as complement activation, can trigger TF synthesis in intravascular cells, such as monocytes and endothelial cells.^26^ As a result of inflammation, endothelial dysfunction and vascular injury may occur leading to rapid generation of thrombin at such sites by activating all the arms of the coagulation system.^27^

CRP is a hallmark of inflammation and serves as a surrogate marker for the inflammatory activity in many rheumatic disorders. Many disease activity formulas take in account the level of CRP as a contributor to the overall disease activity. Several observations even relate a direct pathogenic role to CRP in the process of joint erosions and bone destruction in RA. CRP was shown to be an inducer of the receptor activator of nuclear factor kappa-B ligand (RANKL). It has a direct effect on the differentiation of osteoclast precursors into mature osteoclasts. Therefore, lowering CRP levels is pertinent to the control and prevention of further joint damage in RA patients.^28^ Peters et al.^29^ reported that thrombin-activatable fibrinolysis inhibitor levels were significantly higher in RA patients with a high inflammatory state (CRP >10 mg/L) compared to those with lower CRP levels (CRP <10 mg/L). These reports are in line with our findings that demonstrated the higher probability for VTE in RA patients who have higher CRP levels.

In conclusion, our study has shown that RA is independently associated with VTE. CRP levels were also found to be directly related with an increased risk for VTE. Our results challenge the current understating of this linkage; suggesting that RA should be more commonly known as a risk factor for VTE. This may modify the current manner by which we calculate VTE risk and the need for thromboprophylaxis, as well as our VTE treatment regimens, with a possible effect on the intensity and length of therapy.

## ABBREVIATIONS:

RA: Rheumatoid Arthritis
BMI: Body Mass Index
CHS: Clalit Health Services
CRP: C-Reactive Protein
DVT: Deep Vein Thrombosis
IL-6: Interleukin-6
PE: Pulmonary Embolism
RANKL: Receptor Activator of Nuclear Factor Kappa-B Ligand
RF: Rheumatoid Factor
SES: Socioeconomic Status
TF: Tissue Factor
Tnfα: Tumor Necrosis Factor-Alpha
VTE: Venous Thromboembolism
